# Transcriptomic analysis of early B-cell development in the chicken embryo

**DOI:** 10.3382/ps/pez354

**Published:** 2019-06-25

**Authors:** Nikhil K Nuthalapati, Jeffrey D Evans, Robert L Taylor, Scott L Branton, Bindu Nanduri, Gregory T Pharr

**Affiliations:** 1 Department of Basic Sciences, College of Veterinary Medicine, Mississippi State University, Mississippi State MS 39762, Mississippi State; 2 USDA, Agricultural Research Service, Poultry Research Unit, PO Box 5367, Mississippi State University, Starkville, MS 39762-5367; 3 Division of Animal and Nutritional Sciences, West Virginia University, Morgantown, WV 26506

**Keywords:** transcriptomics, bursa of Fabricius, B-cell development, chicken embryo

## Abstract

The chicken bursa of Fabricius is a primary lymphoid tissue important for B-cell development. Our long-term goal is to understand the role of bursal microenvironment in an early B-cell differentiation event initiating repertoire development through immunoglobulin gene conversion in the chick embryo. We hypothesize that early bursal B-cell differentiation is guided by signals through cytokine receptors. Our theory is based on previous evidence for expression of the receptor tyrosine kinase superfamily members and interleukin receptors in unseparated populations of bursal B-cells and bursal tissue. Knowledge of the expressed genes that are responsible for B-cell differentiation is a prerequisite for understanding the bursal microenvironment's function. This project uses transcriptomic analysis to evaluate gene expression across early B-cell development. RNA-seq was performed with total RNA isolated from bursal B-cells at embryonic day (ED) 16 and ED 19 (n = 3). Approximately 90 million high-quality clean reads were obtained from the cDNA libraries. The analysis revealed differentially expressed genes involved in the *Jak-STAT* pathway, *Wnt* signaling pathway, MAPK signaling pathway, metabolic pathways including tyrosine metabolism, Toll-like receptor signaling pathway, and cell-adhesion molecules. The genes predicted to encode surface receptors, signal transduction proteins, and transcription factors identified in this study represent gene candidates for controlling B-cell development in response to differentiation factors in the bursal microenvironment.

## INTRODUCTION

Humoral immunity is mediated by B-cells that mature in the chicken bursa of Fabricius, whose function was revealed in several elegant experiments conducted (Glick et al., 1956; Cooper et al., 1965, 1966). The bursa, a cloacal appendage, is a primary lymphoid tissue that is important for studying B-cell development (Glick 1988). Chicken B-cell development has been described according to the ability of various B stem cells to restore humoral immunity via adoptive transfer of bursal cells to immunodeficient syngeneic hosts made by irradiation (Lassila et al., 1988) or by treatment with cyclophosphamide (Glick, 1971; Linna et al., 1972). Treatment with irradiation (Lassila et al., 1988) or cyclophosphamide (Glick, 1971; Linna et al., 1972) depletes bursal B-cells while leaving the stroma of the bursal follicles intact. The phenotypic and functional studies conducted by others suggest that at least 3 B-cell stages can be identified, the prebursal, bursal, and postbursal stem cells (Masteller et al., 1997).

In the embryonic period, prebursal stem cells expressing the sialyl Lewis X (sLex) carbohydrate epitope and surface IgM enter the epithelium of the bursal anlage and proliferate, forming the nascent lymphoid follicles between embryonic days (**ED**) 8 to 14 (Masteller et al., 1995a,b). A differentiation event by ED15–17 initiates the process of immunoglobulin (**Ig**) heavy- and light-chain diversification by Ig–gene conversion (Masteller et al., 1997). The onset of Ig–gene conversion has been shown to occur with a change in developing B-cell surface expression of sLex to a structurally similar carbohydrate termed Lewis X (Lex) (Masteller et al., 1995a,b). In functional studies, developing B-cells expressing sLex or Lex were isolated from the bursa at ED15/16 or ED17/18, respectively. The rearranged Ig light-chain gene was then PCR amplified for both populations. The DNA sequence of the PCR-amplified Ig light chain showed that Ig-gene conversion was initiated in 76% of the Lex population, but in only 29% of the sLex cells over 4 independent experiments (Masteller et al., 1995b). Therefore, both phenotypic and functional studies identified an important role for the embryonic bursa in the onset of repertoire development through a major B-cell differentiation event on or about ED17/18 (Masteller et al., 1997).

The long-term goal of our laboratory is to understand the role of bursal microenvironment in directing an early B-cell differentiation event initiating repertoire development through Ig–gene conversion in the chick embryo. To that end, we propose to identify the genes which control the first major B-cell differentiation event in the embryonic period leading to the onset of repertoire development. This knowledge is required to predict the contribution of the bursal microenvironment's differentiation factors that influence expression of genes controlling B-cell differentiation. Therefore, the objective of this work was to conduct RNA sequence analysis of bursal B-cells during ED16 and ED19 to identify differentially expressed genes between 2 developmental time points.

## MATERIAL AND METHODS

### Sample Preparation

Hy-Line W-36 commercial layer fertile eggs were used as the source of embryos. The bursas were collected and B-cells were isolated from embryos at ED 16 and ED 19. We had 3 biological replicates for ED 16 and ED19 each and 55 bursas were pooled for each biological replicate. In brief, bursae were collected and cut into small pieces with scissors. Single-cell suspensions were filtered through gauze pads and the lymphocytes isolated by centrifugation on Histopaque (Sigma-Aldrich, St. Louis, MO, USA).

### RNA Extraction and cDNA Library Preparation

Using Qiagen RNeasy Mini Kit, we isolated total RNA as per the manufacturer's instructions. The RNA samples were evaluated on agarose gels and the purity was checked using a NanoPhotometer spectrophotometer (Implen, Westlake Village, CA, USA). RNA concentrations were determined using the Qubit RNA Assay Kit in Qubit 2.0 Fluorometer (Life Technologies, CA, USA). The RNA integrity was determined using the Agilent 2100 Bioanalyzer (Agilent Technologies, Santa Clara, CA, USA). The RNA integrity average score was 8.8 for ED 16 and 10 for ED 19.

### cDNA Library Preparation RNA Extraction and Sequencing

The library construction and sequencing was conducted at Novogene, Inc. (Chula Vista, CA, USA). Briefly, mRNA was selected with poly-T oligo-attached magnetic beads from 3 μg of total RNA per biological replicate, and then used for library preparation with the NEBNext Ultra RNA Library Prep Kit for Illumina (NEB, Ipswich, MA, USA). Index codes were added in order to assign sequences to each replicate. Briefly, the mRNA was fragmented and then strand cDNA synthesis was primed with random hexamers. After second strand cDNA synthesis, the transcripts were poly-A tailed for ligation of sequencing adaptors. The library fragments were size-selected for cDNA fragments of 150∼200 bp in length, and the library was amplified by PCR. The index-coded samples were sequenced on an Illumina Hiseq platform to generate 125/150 bp paired-end reads. First, raw data (raw reads) of fastq format were processed through in-house perl scripts. In this step, clean data (clean reads) were obtained by removing reads from raw data that were low quality as well as those containing adapter or poly-N. The GC content plus the Q20 and Q30 values were calculated. All analyses were conducted with clean data reads. The galGal4 chicken genome assembly was used as the reference genome for alignments with paired-end clean reads using TopHat v2.0.12 (ftp://ftp.ensembl.org/pub/release81/fasta/gallus_gallus/dna/Gallus_gallus.Galgal4.dna.toplevel.fa.gz). The data are accessible through NCBI GEO accession number GSE131652.

### Gene Expression Analysis

The HTSeq v0.6.1 software was used to determine the number of reads mapped to each gene. The fragments per kilobase of transcript sequence per million of reads mapped (**FPKM**) was calculated. From the TopHat alignment results, the Cufflinks v2.1.1 Reference Annotation Based Transcript assembly method was used to identify the known and novel transcripts. Asprofile v1.0 was used to classify alternative splicing (**AS**) events to 12 basic types. In each sample, the number of AS events was estimated separately. The DESeq R package (1.18.0) was used to conduct differential expression analysis using negative binomial distribution. The *P*-values that resulted from negative binomial distribution were adjusted with the Benjamini-Hochberg procedure. Genes with an adjusted *P* value < 0.05 were judged to be differentially expressed.

The reproducibility of the biological replicates was determined by calculating the squared Pearson correlation coefficient using the Log2 of expression values with the EdgeR package (3.0.8) (Robinson et al., 2010). The Gene ontology (**GO**) enrichment analysis of differentially expressed genes was conducted with the GOseq R package by correcting the gene length bias. A corrected *P* value < 0.05 indicated significantly enriched GO terms. The Kyoto Encyclopedia of Genes and Genomes (**KEGG**) database was used to interpret the biological functions of differentially expressed genes. A statistical enrichment test was performed with the KOBAS software for the differentially expressed genes predicted in KEGG pathways.

## RESULTS AND DISCUSSION

### Quality Control Parameters of RNA-Seq Data

To study the B-cell developmental stages at ED16 and ED19, we used RNA-Seq to measure differential gene expression. The Illumina Hiseq platform was used to generate an average of 99,370,712 clean reads for ED16 and 83,276,934 clean reads for ED19 representing an average total of 14.87 and 12.49 G, respectively. There was a very low error rate distribution (Supplementary Figure S1), indicating high-quality library construction. The error rate, Q20, Q30 percentages, Guanine Cytosine (GC) content, and percentage of mapped reads were shown along with the number of clean reads with biological replicates is shown in Table 1.

**Table 1.** Statistics of sequencing data for embryonic days 16 and 19 bursal cells from Hy-Line W-36 embryos.

Sample name	Raw reads	Clean reads	Clean bases	Error rate (%)	Q20 (%)	Q30 (%)	GC content (%)
ED16–1	110,869,954	108,265,978	16.24G	0.02	95.65	89.81	54.64
ED16–2	93,359,088	91,273,790	13.69G	0.02	95.70	89.83	53.61
ED16–3	104,939,382	98,572,368	14.79G	0.02	94.88	88.45	54.55
ED19–4	97,987,760	93,731,648	14.06G	0.02	96.01	90.45	47.75
ED19–5	84,679,282	81,008,984	12.15G	0.02	95.69	89.70	47.76
ED19–6	78,646,522	75,090,170	11.26G	0.02	95.85	90.13	47.90

ED: embryonic day.

(1) Sample name: the names of samples.

(2) Raw Reads: the original sequencing reads counts.

(3) Clean Reads: number of reads after filtering.

(4) Clean Bases: clean reads number multiply read length, saved in G unit.

(5) Error Rate: average sequencing error rate, which is calculated by Qphred = –10log10€.

(6) Q20: percentages of bases whose correct base recognition rates are greater than 99% in total bases.

(7) Q30: percentages of bases whose correct base recognition rates are greater than 99.9% in total bases.

(8) GC content: percentages of G and C in total bases.

In general, DNA containing rich GC content is considered as “good” DNA (Vinogradov, 2003). The GC content distribution (Supplementary Figure S2) was evaluated to detect potential GC bias, which could affect gene expression quantification. In general, G should be equal to C and T should be equal to A during the sequencing for non-stranded libraries. GC content in a genomic region effects the total number of reads produced during the RNA sequencing (Benjamini and Speed, 2012). In our data set, the GC content is comparable to AT content, indicating that there is no GC bias in gene expression quantification (Supplementary Figure S2).

### Differential Gene Expression between ED16 and ED19 B-cell Developmental Stages

The analyses of differential gene expression were based on clean reads (Supplementary Figure S3). An overview of the mapping status of the 2 bursal B-cell developmental stages with biological replicates plus the percentage of reads mapped are shown in Table 2. Mapped regions are categorized as exons, introns, or intergenic regions (Supplementary Figure S4). To be well annotated for a reference genome, exons should represent the majority of mapped reads. An average of >80% of exon mapped reads in the data sets suggested that the reference genome was well-annotated. In addition, some intron-mapped reads were also found which might be derived from AS or possibly insufficient reference genome annotation. Distribution plots of reads mapped to chromosomes for each sample, where a window size of 1 K was set, show that greater than 75% of the reads mapped to exons (Supplementary Figure S5).

**Table 2.** Overview of Mapping Status for embryonic days 16 and 19 bursal cells from Hy-Line W-36 embryos.

Sample name	ED16–1	ED16–2	ED16–3	ED19–4	ED19–5	ED19–6
Total reads	108,265,978	91,273,790	98,572,368	93,731,648	81,008,984	75,090,170
Total mapped	72,596,169 (67.05%)	63,189,781 (69.23%)	64,549,916 (65.48%)	73,839,829 (78.78%)	63,762,738 (78.71%)	58,442,981 (77.83%)
Multiple mapped	1,294,393 (1.2%)	1,128,156 (1.24%)	881,085 (0.89%)	779,336 (0.83%)	836,206 (1.03%)	614,071 (0.82%)
Uniquely mapped	71,301,776 (65.86%)	62,061,625 (68%)	63,668,831 (64.59%)	73,060,493 (77.95%)	62,926,532 (77.68%)	57,828,910 (77.01%)
Reads map to “+”	35,643,288 (32.92%)	31,017,135 (33.98%)	31,840,274 (32.3%)	36,514,788 (38.96%)	31,445,453 (38.82%)	28,903,917 (38.49%)
Reads map to “–”	35,658,488 (32.94%)	31,044,490 (34.01%)	31,828,557 (32.29%)	36,545,705 (38.99%)	31,481,079 (38.86%)	28,924,993 (38.52%)
Non-splice reads	38,260,637 (35.34%)	33,575,763 (36.79%)	33,505,632 (33.99%)	41,264,205 (44.02%)	34,804,685 (42.96%)	31,734,121 (42.26%)
Splice reads	33,041,139 (30.52%)	28,485,862 (31.21%)	30,163,199 (30.6%)	31,796,288 (33.92%)	28,121,847 (34.71%)	26,094,789 (34.75%)

Mapping Results Details:

1. Total number of filtered reads (Clean data).

2. Total number of reads that can be mapped to the reference genome. In general, this number should be larger than 70% when there is no contamination and the correct reference genome is chosen.

3. Number of reads that can be mapped to multiple sites in the reference genome. This number is usually less than 10% of the total.

4. Number of reads that can be uniquely mapped to the reference genome.

5. Number of reads that map to the positive strand (+) or the minus strand (–).

6. Splice reads can be segmented and mapped to 2 exons (also named junction reads), whereas non-splice reads can be mapped entirely to a single exon. The ratio of splice reads depends on the insert size used in the RNA-Seq experiments.

### Alternative Splicing

Alternative splicing, where exons of pre-mRNAs from genes can be spliced differently to produce both functionally and structurally distinct mRNA (Blencowe, 2006), is universal in gene regulation. More than 90% of human genes display AS (Croft et al., 2000; Pan et al., 2008). The biological function of AS is to effectively increase the coding of the genome (Mockenhaupt and Makeyev, 2015). The replicate multivariate analysis of transcript splicing was used to identify differential AS from RNA-Seq data. There are 5 types of AS events identified by replicate multivariate analysis of transcript splicing that include, skipped exon (SE), alternative 5′ splice site (A5SS), alternative 3′ splice site (A3SS), mutually exclusive exons (MXE), and retained intron (RI) (Supplementary Figure S6). The classification and statistical analysis of AS events revealed that SE and MXE were the predominant AS types identified in the current study as shown in Supplementary Figure S7 and the statistics of AS events is shown in Table 3.

**Table 3.** Statistics of alternative splicing (AS) events for embryonic days 16 and 19 bursal cells from Hy-Line W-36 embryos.

Event type	NumEvents.JC.only	SigEvents.JC.only	NumEvents.JC+readsOnTarget	SigEvents.JC+readsOnTarget
SE	17,836	300 (91:209)	17,848	322 (98:224)
MXE	2,802	852 (494:358)	2,803	854 (489:365)
A5SS	6	0 (0:0)	6	0 (0:0)
A3SS	17	1 (0:1)	17	1 (0:1)
RI	12	0 (0:0)	12	0 (0:0)

(1) event_type: AS event types (SE, MXE, A5SS, A3SS, RI).

(2) NumEvents.JC.only: the total number of AS events, with only reads span splicing junctions taken into account.

(3) SigEvents.JC.only: the total number of differential AS events, with only reads span splicing junctions taken into account(up:down).

(4) NumEvents.JC+readsOnTarget: the total number of AS events, with both reads span splicing junctions and reads on target exons taken into account.

(5) SigEvents.JC+readsOnTarget: the total number of differential AS events, with both reads span splicing junctions and reads on target exons taken into account.

**Figure 1.**
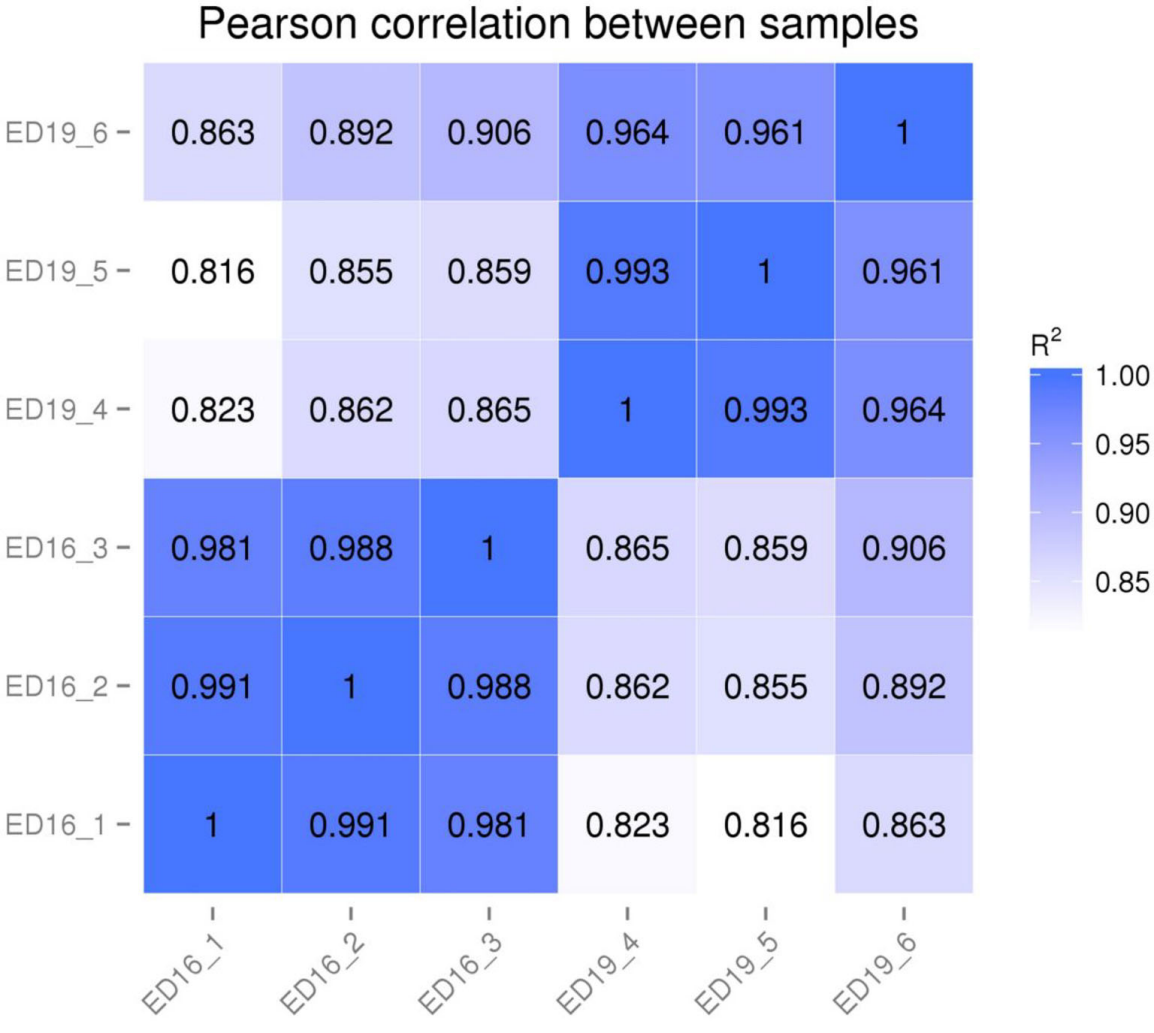
RNA-Seq correlation for embryonic days 16 and 19 bursal cells from Hy-Line W-36 embryos. RNA-Seq correlation: heat maps of the correlation coefficient between samples are shown.

Three biological replicates were conducted at ED16 and ED19 (Hansen et al., 2011). Gene expression levels between replicates were correlated using the squared Pearson correlation coefficient (R^2^) [Figure 1]. In the experiments, the R^2^ value was greater than 0.8. The transcript abundance was used to estimate the gene expression level. The RPKM (Reads per Kilo bases per Million reads) method (Mortazavi et al., 2008) was applied to make the gene expression level estimates comparable among different genes and experiments. The calculated gene expression level was used for comparing the gene expression differences among samples (Table 4).

**Table 4.** The number of genes with different expression levels for embryonic days 16 and 19 bursal cells from Hy-Line W-36 embryos.

FPKM interval	ED16_1	ED16_2	ED16_3	ED19_4	ED19_5	ED19_6
0∼1	7,904 (41.13%)	7,923 (41.22%)	8,307 (43.22%)	8,650 (45.01%)	8,800 (45.79%)	9,310 (48.44%)
1∼3	2,440 (12.70%)	2,366 (12.31%)	2,324 (12.09%)	1,676 (8.72%)	1,623 (8.44%)	1,631 (8.49%)
3∼15	4,159 (21.64%)	4,126 (21.47%)	3,935 (20.47%)	3,485 (18.13%)	3,456 (17.98%)	3,484 (18.13%)
15∼60	2,940 (15.30%)	3,070 (15.97%)	2,878 (14.97%)	3,703 (19.27%)	3,652 (19.00%)	3,039 (15.81%)
>60	1,776 (9.24%)	1,734 (9.02%)	1,775 (9.24%)	1,705 (8.87%)	1,688 (8.78%)	1,755 (9.13%)

FPKM, fragments per kilobase of transcript sequence per million of reads mapped.

The table shows gene count table of different expression levels and calculation of gene numbers in different expression levels.

**Figure 2.**
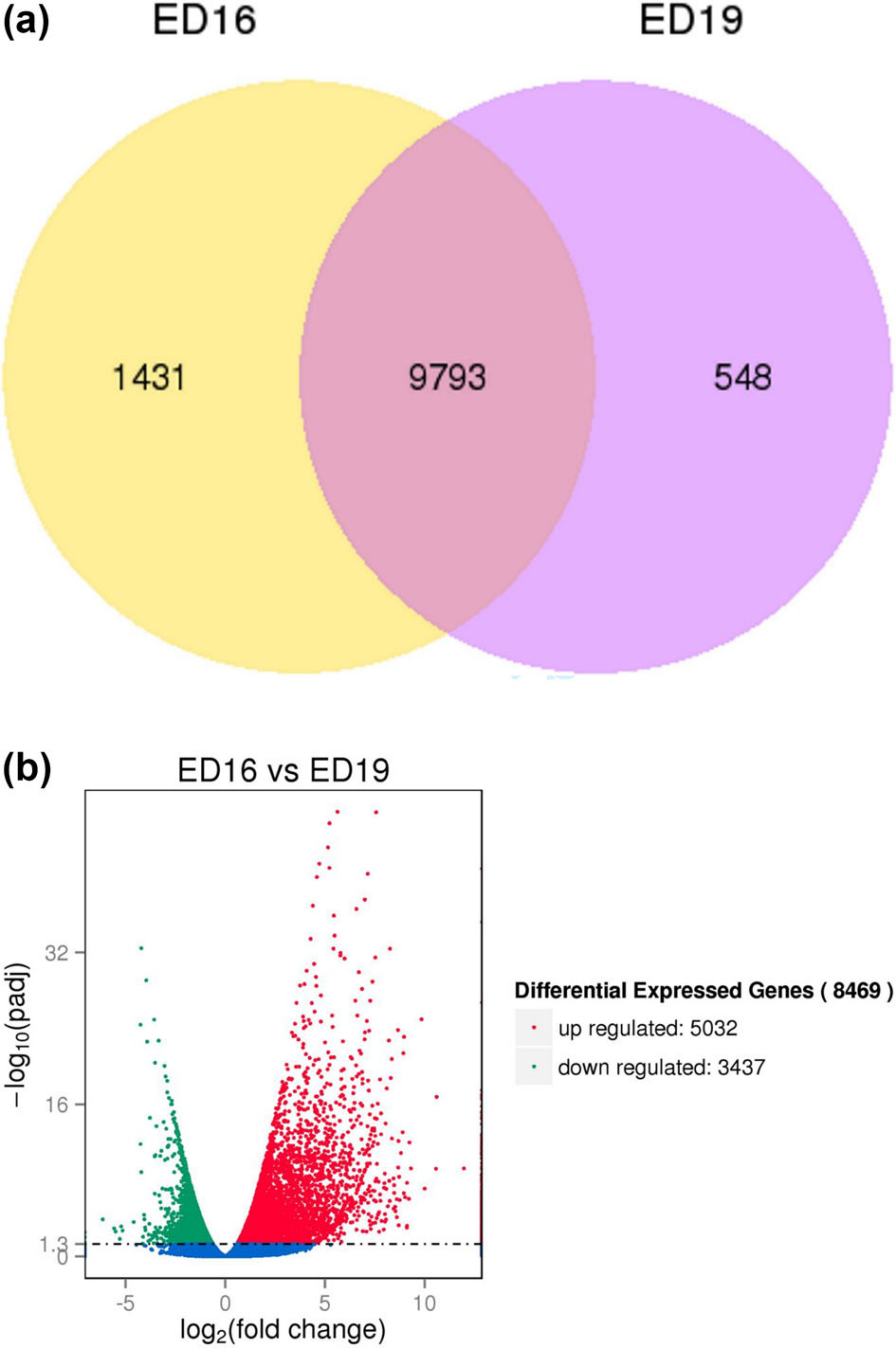
(**a**) Venn diagram of expressed genes for embryonic days (ED) 16 and 19 bursal cells from Hy-Line W-36 embryos. Venn diagram of expressed genes between ED16 and ED19 is shown here. The yellow portion (left) represents the number of genes expressed during ED16, the purple portion (right) represents the number of genes expressed during ED19, and the middle portion represents the genes that were expressed during both ED16 and ED19. (**b**) Volcano plot for differentially expressed genes for ED16 and ED19 bursal cells from Hy-Line W-36 embryos. The x-axis shows the fold change in gene expression between different samples, and the y-axis shows the statistical significance of the differences. Significantly up- and downregulated genes are highlighted in red and green, respectively. Genes did not express differently between ED16 and ED19 are in blue.

For a comparison of gene expression levels at different days of embryonic development, an FPKM distribution was calculated with density plot (Supplementary Figure S8) between ED16 (pink) and ED19 (blue). Supplementary Figure S8 shows that a larger number of genes have a similar distribution of RNA-seq read counts. Moreover, a violin plot representation of the data suggests that the 2 groups (ED16 and ED19) were almost uniform in gene density (Supplementary Figure S9). There are 11,772 genes expressed during ED16 and ED19. From that total, only 1,431 were expressed during ED16 and only 548 during ED19. There were 9,793 genes were expressed during both ED16 and ED19 (Figure 2a).

The DESeq R package was used for detection of differentially expressed genes by adding 3 biological replicates which was considered more appropriate than increasing the sequence depth to reduce the number of false positives. In general, a continuous distribution will not be observed for read count data in RNA-Seq or microarray analysis. The Poisson distribution, a common procedure in prior times, is not optimal for RNA-Seq data because it considers mean and variance to be equal (Bullard et al., 2010; Jiang et al., 2011; Anders and Huber, 2010). Furthermore, Poisson distribution can create overdispersion with biological replicates of RNA-Seq read count data. The negative binomial distribution which uses the generalized linear model and revamps to multifactor comparisons by DESeq was used to read the count data in the present study (Anders and Huber, 2010; Robinson et al., 2010; McCarthy et al., 2012). Mean and variance are not treated as equal in negative binomial distribution, and the analysis depends on estimated dispersion parameter controlling the correlation between mean and variance of read count data.

The Volcano plot analysis was used to depict the general distribution of differentially expressed genes (Figure 2b). Of the 8,469 genes that were differentially expressed, 5,032 genes were upregulated and 3,437 genes were downregulated. The upregulated differentially expressed genes and downregulated differentially expressed genes are attached as supplement excel files (S-Up-regulated Differentially Expressed Genes excel file and S-Down-regulated Differentially Expressed Genes excel file, respectively). Hierarchical cluster analysis was used to group differentially expressed genes into clusters based on function or under the same biological process. The analysis revealed genes that were highly expressed at the 2 developmental time points, suggesting that these genes with specific functions may be more related to biological characteristics of ED16 and ED19 stages of bursal B-cell development (S-Heat Cluster Detail).

### Gene ontology and KEGG Pathway Enrichment Analysis of Differentially Expressed Genes (DEGs)

**Figure 3.**
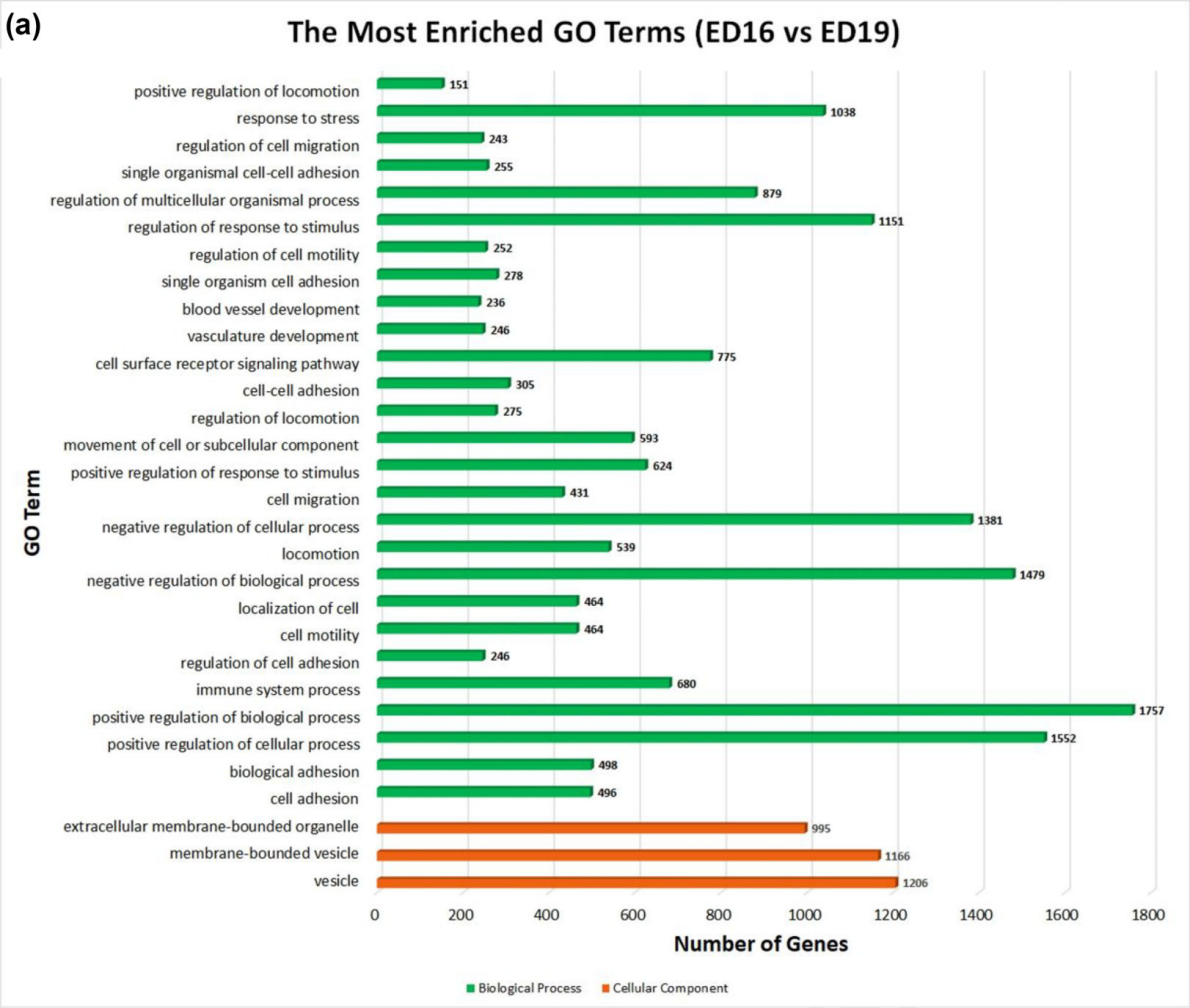

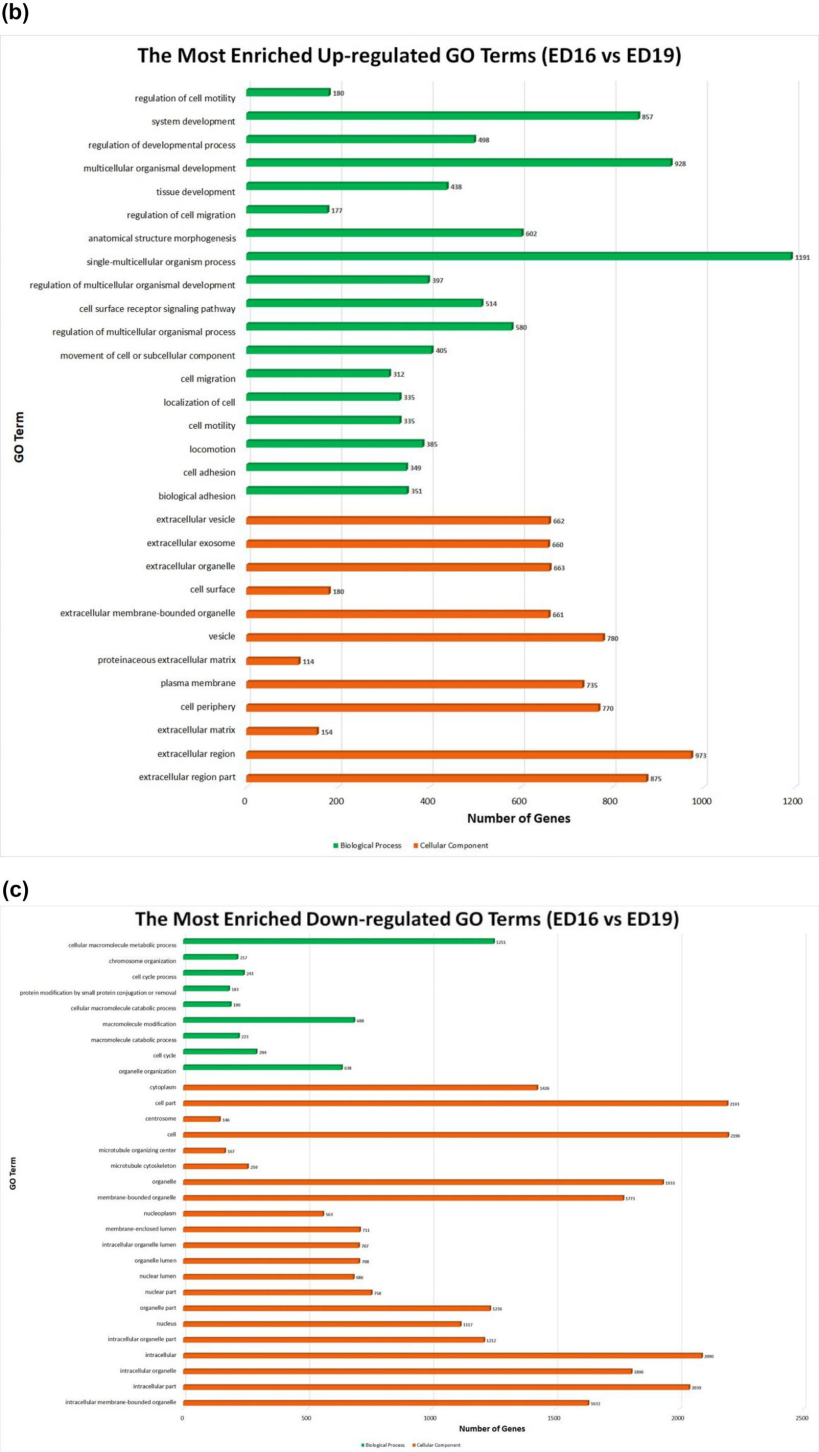

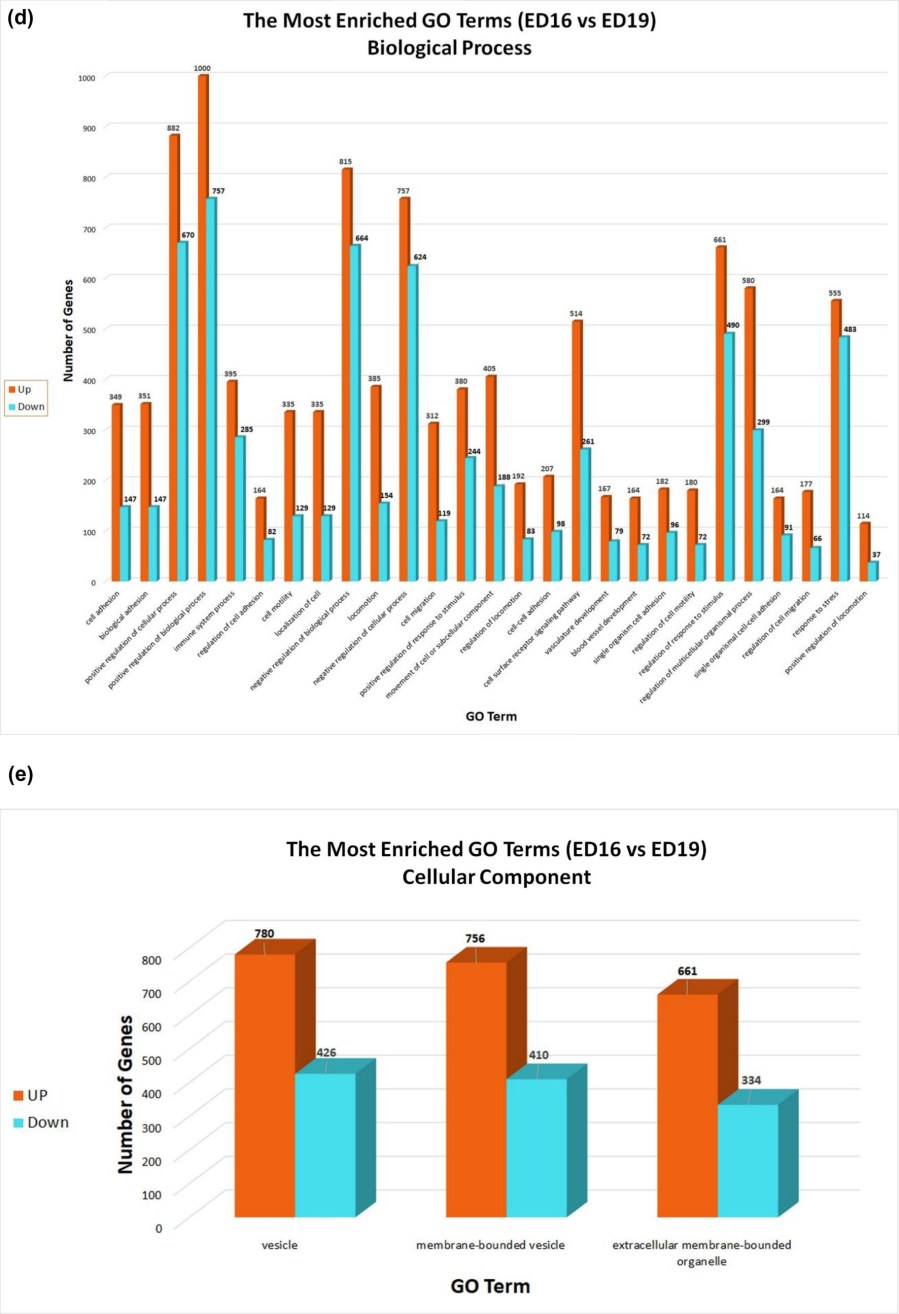
Gene ontology enrichment bar chart of DEGs for embryonic days 16 and 19 bursal cells from Hy-Line W-36 embryos. (**a**) The most enriched GO terms. **(b**) The most enriched upregulated GO terms. (**c**) The most enriched downregulated GO terms. (**d**) The most enriched biological process up-down GO terms. (**e**) The most enriched cellular component up-down GO terms. GO enrichment bar chart of DEGs: the GO enrichment bar chart of DEGs presents the number of DEGs enriched in biological process, cellular component, and molecular function. The 30 most significant enriched terms are selected. (**a**) The y-axis is the enriched GO term; x-axis is the number of DEGs enriched in this term. Colors represent different GO types: biological process (green color), cellular component (orange color), and molecular function. (**b**) The y-axis is the enriched upregulated GO term; x-axis is the number of DEGs enriched in this term. Colors represent different GO types: biological process (green color), cellular component (orange color), and molecular function. (**c**) The y-axis is the enriched downregulated GO term; x-axis is the number of DEGs enriched in this term. Colors represent different GO types: biological process (green color), cellular component (orange color), and molecular function. (**d**) The y-axis is the enriched GO term for biological process; x-axis is the number of DEGs enriched in this term. Colors represent up- and downregulation. (**e**) The y-axis is the enriched GO term for cellular component; x-axis is the number of DEGs enriched in this term. Colors represent up- and downregulation.

The GO terms are widely used to describe cellular components, molecular functions, and biological process of genes. A GO enrichment bar chart is used to illustrate the enriched GO terms and the counts of DEGs for each GO term. Examination of the most enriched 30 GO annotation terms (Figure 3a) revealed significant DEGs enrichment in the following categories of cell adhesion, detection, and response to stimulus/stress, development of blood vessel, vasculature, membrane proteins, locomotion, vesicle, regulation of immune system process and positive/negative regulations of biological process and cellular process.

The 30 most enriched upregulated GO terms (Figure 3b) revealed that the DEGs are significantly enriched in the classification of development process, regulations, cell adhesion in biological process, and a large number of GO terms of membranes and envelopes are observed in cellular components. The 30 most enriched downregulated GO terms (Figure 3c) revealed that the DEGs are most significantly enriched in the cell cycle, metabolic process, and chromosome organization in biological process with a large number of GO terms of cell cycle components, membrane proteins, and organelle membranes and envelopes are observed in cellular components.

Additionally, the most enriched Biological Process up-down GO terms (Figure 3d) revealed that there are more upregulated DEGs than downregulated DEGs in the Biological Processes. The most enriched Cellular Components of up-down GO terms (Figure 3e) revealed that the DEGs are mostly upregulated than down-regulated in categories of vesicles, membrane and envelopes of Cellular components ontology.

**Figure 4.**
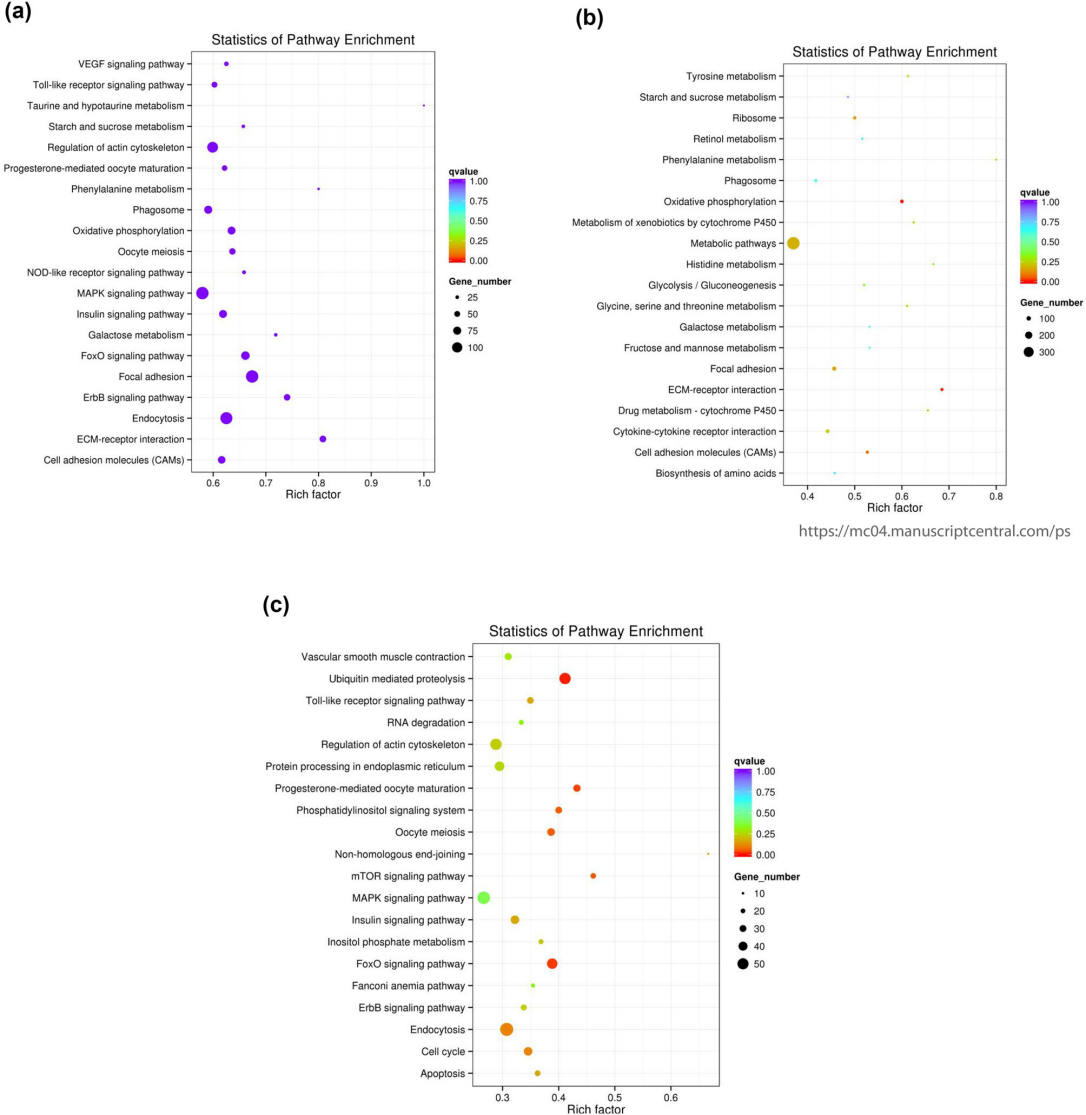
KEGG enrichment scattered plot for embryonic days 16 and 19 bursal cells from Hy-Line W-36 embryos. (**a**) DEG-enriched KEGG pathway scatterplot. (**b**) Upregulated DEG-enriched KEGG pathway scatterplot. (**c**) Downregulated DEG-enriched KEGG pathway scatterplot. The KEGG enrichment scatter plot: the most significant enriched 20 pathways are presented in the scatter plot. (**a**) The scattered plot of all DEGs: x-axis represents the name of the pathway and the y-axis represents the rich factor. The size stands for the number of difference genes and the color stands for different Q-values. (**b**) The scattered plot of upregulated DEGs: x-axis represents the name of the pathway and the y-axis represents the rich factor. The size stands for the number of difference genes and the color stands for different Q-values. (**c**) The scattered plot of downregulated DEGs: x-axis represents the name of the pathway and the y-axis represents the rich factor. The size stands for the number of difference genes and the color stands for different Q-values.

The DEGs were then mapped to KEGG terms to understand if the genes with predicted functions in different biological pathways were significantly enriched. The KEGG enrichment analysis results are displayed as a scatter diagram in Figure 4a–c. In the analysis plot, the level of KEGG enrichment is measured through the rich factor, Q-value, and gene counts enriched in a particular pathway. The rich factor is the ratio of the DEGs to all annotated genes in a particular pathway. A large rich factor reflects a higher level of enrichment. Q-value is the adjusted *P* value after multiple testing with a range of 0 to 1. A level of KEGG enrichment is indicated as the Q-value approaches zero.

Our KEGG enriched scatter plot analysis revealed that 5,005 DEGs were assigned to 154 pathways (S-Kegg Scatter Plot Analysis -Sheet-All), for DEG enriched KEGG scatter plot, 3,044 upregulated DEGs were assigned to 153 pathways (S-Kegg Scatter Plot Analysis Up-Regulated), and 1,961 downregulated DEGs were assigned to 143 pathways (S-Kegg Scatter Plot Analysis Down-Regulated). The top 20 most significant enriched pathways were chosen for total (Figure 4a), upregulated (Figure 4b), and downregulated (Figure 4c) DEGs enriched KEGG pathway scatter plots.

This study evaluated gene expression in early B-cell development using transcriptomic analysis of bursal B-cells. Previous studies from this lab produced evidence for expression of interleukin receptors and receptor tyrosine kinase superfamily members in unseparated populations of bursal B-cells and bursal tissue (McCarthy et al., 2006; Felfoldi et al., 2008; Pharr et al., 2009). These data led to the hypothesis that signals through cytokine receptors could be important for the early B-cell differentiation event(s) in the embryonic bursa. The hypothesis further predicted that genes showing differential expression between the 2 developmental time points may decide cell fate such as survival, proliferation, and differentiation. Therefore, based on the hypothesis, ED16 and ED19 cell surface protein-encoding genes associated with the pathways JAK-STAT, Wnt signaling, and MAPK signaling identified with KEGG analysis (Table 5) are discussed below.

**Table 5.** Genes encoding surface proteins predicted to be associated with pathways identified with KEGG enrichment analysis in embryonic days 16 and 19 bursal cells from Hy-Line W-36 embryos.

	Gene ID	Log fold change	Gene name
**MAP kinase Pathway**	ENSGALG00000012357	–1.14700	CXCR4
	ENSGALG00000007675	–0.45604	CXCR5
**JAK-STAT Pathway**	ENSGALG00000005638	0.86514	CD132
	ENSGALG00000006313	–0.66557	IL-4RA
	ENSGALG00000014716	–0.46118	Gp130
**Wnt Pathway**	ENSGALG00000009064	0.20678	FZD1
	ENSGALG00000016627	0.34486	FXD3
	ENSGALG00000016069	0.39605	FZD 6
	ENSGALG00000023536	5.54310	FZD7
	ENSGALG00000028487	2.79460	FZD8
	ENSGALG00000006998	–0.07460	LR5
	ENSGALG00000011558	–1.25180	LRP6
	ENSGALG00000006488	–0.76496	RYK

The table shows the genes along with gene ID, its Entrez gene name, and the fold change in our experiment and what pathway they are associated with.

### JAK-STAT Signaling Pathway

The chicken IL-4 receptor alpha and the signal transducing subunit CD132 (Table 5) represent candidates for activating the JAK-STAT pathway in bursal B-cells, leading to the activation of transcription factors that may induce B-cell differentiation. The expression of the chicken IL-4 receptor in the bursa has been found at the protein (McCarthy et al., 2006) and cDNA level (Caldwell et al., 2005; Liu et al., 2016; DT40 RNA-seq expression data GSE58766, Smith et al., 2015).

The IL-4 receptor activates JAK1 and JAK2 which then phosphorylate STAT6 (Mokada-Gopal et al., 2017). Phosphorylated STAT6 proteins form homodimers and translocate to the nucleus transactivating genes associated with cell proliferation and events associated with germinal centers. Recent mammalian studies have shown that STAT6 is critical for the development of germinal centers. Germinal centers are responsible for somatic hypermutation to improve the B-cell receptor affinity for the antigen epitope, and H-chain class switching, both of which are mediated by the product of the activation-induced cytidine deaminase (**AICDA**) gene. In STAT6-deficient B-cells, a number of genes important for germinal centers were downregulated, including the major transcription regulator Bcl6 (Turqueti-Neves et al., 2014).

Two major transcriptional regulators control B-cell biology in the germinal center, Bcl6, and Bach2. These 2 genes have opposing functions that regulate germinal center B-cells. Bcl6 works as a transcriptional repressor to prevent expression of DNA-damage response genes to allow B-cells to proliferate and survive while undergoing somatic hypermutation and double-strand DNA breaks due to class switching. Bach2, on the other hand, is activated by signals through the B-cell receptor and IL-7 receptor (Tamahara et al., 2017) to stimulate both cell cycle checkpoint genes as well as a cell death program due to DNA damage (Swaminathan et al., 2014). Therefore, the cooperation between the 2 major transcriptional regulators is postulated to ensure proper affinity maturation and differentiation in the germinal center (Huang et al., 2014).

High levels of Bcl6 transcripts at ED16 and ED19 were found as was a 2-fold increase in Bach2 transcripts in ED19 (S-Differentially Expressed Genes). With relevance to our work, Bcl6 is one of the many genes induced by IL-4 receptor/STAT6 signaling in mammals (Ruiz-Lafuente et al., 2014; Turqueti-Neves et al., 2014). Recent studies demonstrate a possible role for Bcl6 and Bach2 in chicken repertoire development. The disruption of the Bcl6 gene in the chicken bursal B-cell line DT40 eliminated Bach2 and AICDA transcripts. Additional experiments showed that Bcl6 transactivates Bach2 and targets the AICDA gene in cooperation with Pax5 (Alinikula et al., 2011). While Bcl6 not only transactivates AICDA, the activation of additional genes by Bcl6 are also required for Ig–gene conversion (Williams et al., 2016). Moreover, a Bach2 deficiency in the same cell line abrogated Ig–gene conversion and showed that Bach2 may transactivate the AICDA promoter as well as transactivate genes associated with function of the AID protein (Budzynska et al., 2017). Therefore, it may be possible that IL-4 could represent one of the microenvironmental signals during embryonic bursal B-cell development which supports the expression of transcriptional regulators associated with Ig–gene conversion.

The gp130 represents the signal transducing subunit for IL-6 cytokine family receptors. The signal through JAK1 activates STAT genes, STAT3, the predominant form as well as STAT1 and STAT5 (Hirano et al., 2000). The levels of gp130 transcripts did not differ between ED16 and ED19. Those gene transcripts have also been reported in the DT40 database, in the posthatch bursa (Sun et al., 2015; Liu et al., 2016), and at the protein level in the posthatch bursa (McCarthy et al., 2006). We found only low levels of transcripts for alpha receptor candidates of various IL-6 family cytokines, leukemia inhibitory factor, oncostatin, and ciliary neurotrophic factor receptors (S-Differentially Expressed Genes). An IL-11 receptor alpha candidate which is expressed in the posthatch bursa (McCarthy et al., 2006; Sun et al., 2015; Liu et al., 2016) and Harderian gland (Deist and Lamont, 2018) is of interest. In mammals and chickens, IL-11 is a stromal-derived cytokine controlling various biological properties of cells including proliferation, differentiation, and cytokine production (Xu et al., 2016; Truong et al., 2018). Of interest to our work, the combined effect of IL-11 with different hematopoietic cytokines induced the differentiation of both myeloid and lymphoid progenitors in mammalian hematopoiesis (Ray et al., 1996; Weich et al., 2000). In future studies it would be of interest to isolate the follicular reticular epithelial cells for evaluation of cytokine synthesis to identify a source for IL-11 in the bursa.

### Wnt Signaling Pathway

Wnts are secreted cysteine-rich glycosylated and lipid modified proteins (Mills et al., 2017). The Wnt/β-catenin pathway, or so-called canonical Wnt signaling, is well known for a signaling stream that is involved in chicken primordial germ cells development, specification, migration, and proliferation (Kimura et al 2006; Ohinata et al., 2009; Laird et al., 2011; Chawengsaksophak et al., 2012). Recent studies indicate that Wnt ligands and Wnt receptors are expressed in the bursa (Sun et al., 2015; Liu et al., 2016; Monson et al., 2018). In the current study, Frizzled (**FZD**) receptors FZD2, 7, 8, and 10 and Wnt co-receptors low-density lipoprotein-related proteins (**LRP**) LRP1, 2, and 4 were upregulated from ED16–ED19. In mammals, a role for Wnt signaling in lymphopoiesis has been well characterized. The mouse thymus exhibited differential expression of FZD receptors between the different T-cell development stages, whereas Wnt ligands are predominantly secreted by thymic epithelial cells (Weerkamp et al., 2006). Given that contact between bursal B-cells and the follicular epithelial cells is critical for B-cell differentiation (Weber, 2000; Funk and Palmer, 2003), it is reasonable to hypothesize that Wnt ligands could serve as soluble or cell-bound factors that induce bursal B-cell differentiation.

### MAPK Signaling Pathway

Mitogen-activated protein kinase (**MAPK**) signaling pathway is involved in cell proliferation and differentiation. It allows for the environmental adaptation which is necessary for activation and regulation of correlated events important for cell survival (Katz et al., 2007). There are 7 distinct MAPK groups that have been characterized in mammalian cells: extracellular regulated kinases (ERK1/2), c-Jun NH2 terminal kinases (JNK1/2/3), p38 MAPK (p38 α/β/γ/δ), ERK3/4, ERK5, ERK7/8, and Nemo-like kinase (NLK) (Broom et al., 2009; Xing et al., 2010). Substantial studies have been done on ERK1/2, JNKs, and p38 MAPK. Samples from ED16–ED19 showed upregulated MAPK12, MAPK13, MAPKAPK2, MAPKAPK3, MAPK8IP1, MAPK8IP2 and downregulated MAPK1, MAPK6, MAPK9, MAPK11, MAPK14, MAPK8IP3, MAPKAPK5, MAPKBP1, and NLK. Candidates for the MAPK pathway activation that are relevant to our long term interest would include the receptors for CXC chemokines, CXCR4 and CXCR5 (Table 5). The receptors respond to chemoattractant cytokines that direct the migration of leukocytes (Lopez-Cotarelo et al., 2017), and based on mammalian studies, the chemokine receptor transcripts identified here are predicted to encode G-protein coupled receptors activating MAPK signal transduction (Sun et al., 2002). Both CXCR4 and CXCR5 are expressed on bursal B-cells (Koskela et al., 2003; McCarthy et al., 2005; Haertle et al., 2017). Elegant functional studies showed that CXCR5^+^ bursal B-cells migrate in response to recombinant CXCL13 (Haertle et al., 2017). An important role for CXCR4 and CXCR5 has been proposed in the colonization of the embryonic bursa as both chemokine ligands are expressed in bursal tissue (Read et al., 2005; McCarthy et al., 2006). Recruitment of prebursal stem cells from the vasculature into the mesenchyme requires CXCR4, whereas CXCR5 is required for directional migration to the developing bursal follicles (Ratcliffe and Hartle, 2014).

## CONCLUSION

The RNA-Seq data analysis of bursal B-cells at ED16 and ED19 generated over 90 million high-quality clean reads from cDNA libraries using deep RNA sequencing. The DEGs identified in bursal B-cells at 2 developmental time points represent candidates for guiding B-cell differentiation. With increased understanding of the genes involved in B-cell differentiation, it will be possible to predict soluble or surface expressed factors in the embryonic bursa that regulate the expression of these genes, therefore identifying roles for the non-lymphoid cells present in the embryonic bursal follicles. In future studies, we plan to further validate gene expression in the 2 B-cell stages using reverse transcriptase PCR and/or quantitative real-time PCR. These findings will be extended with flow cytometry and western blotting experiments.

## SUPPLEMENTARY DATA

Supplementary data are available at *Poultry Science* online.

pez354_Supplemental_Files
