# Supplementary material for: Transcriptomic analysis of early B-cell development in the chicken embryo
Source: Poult Sci. 2019 Jun 25;98(11):5342–54. doi: 10.3382/ps/pez354 (PMC6771548; doi:10.3382/ps/pez354)
Supplement: pez354_Supplemental_Files [file pez354_supplemental_files.zip › ps-19-08911-S006.docx]

**S-Figure 1: Error rate Distribution**


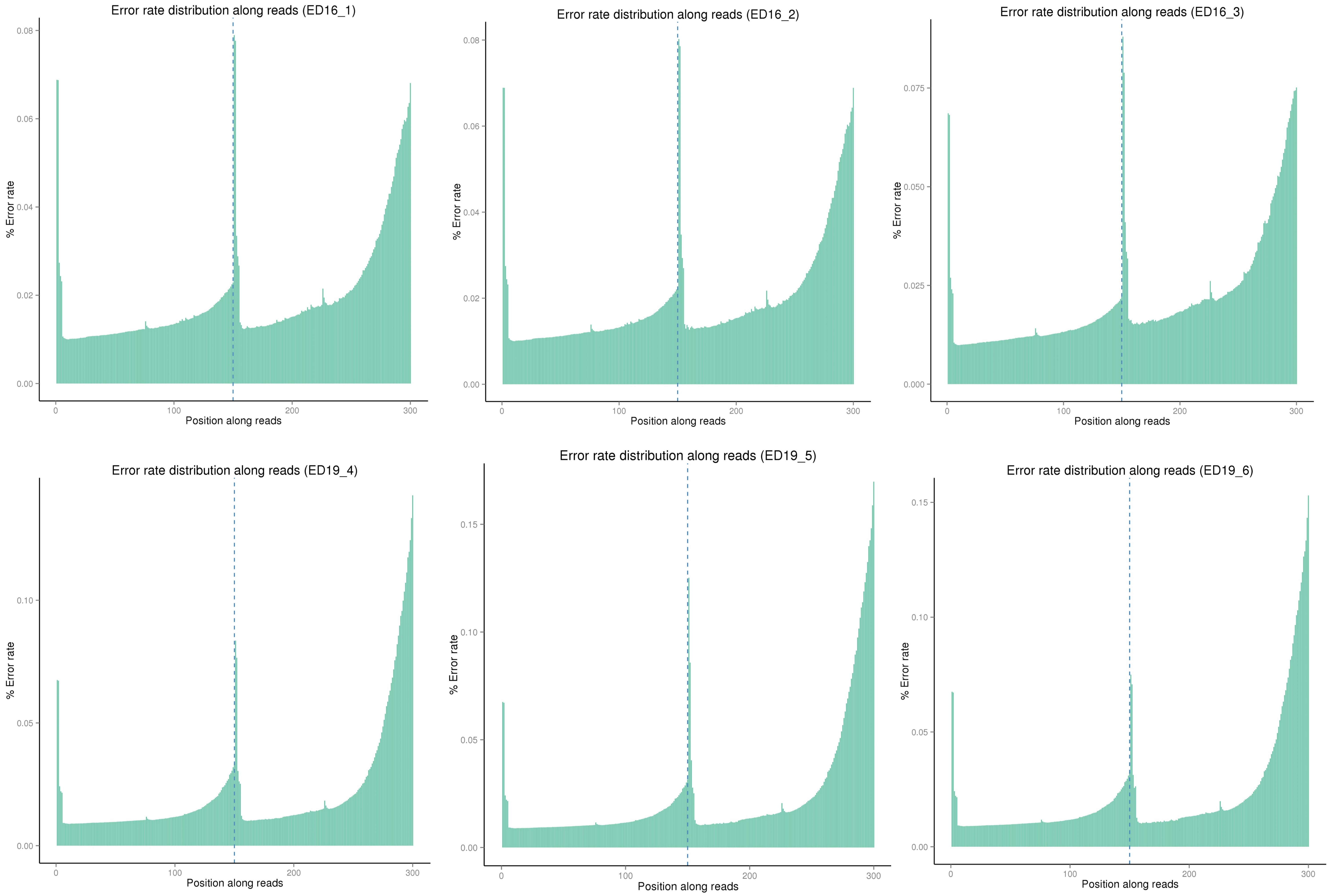


The x-axis shows the base position along each sequencing read and the y-axis shows the base error rate. Sequencing error rate and base quality depend on the sequencing machine, reagent availability, and the samples.

(1) Error rate increases as the sequencing reads are extended and sequencing reagents become more and more scarce.

(2) The first six bases have a relatively high error rate due to the random hexamers used in priming cDNA synthesis.

**S-Figure 2: GC Content Distribution**


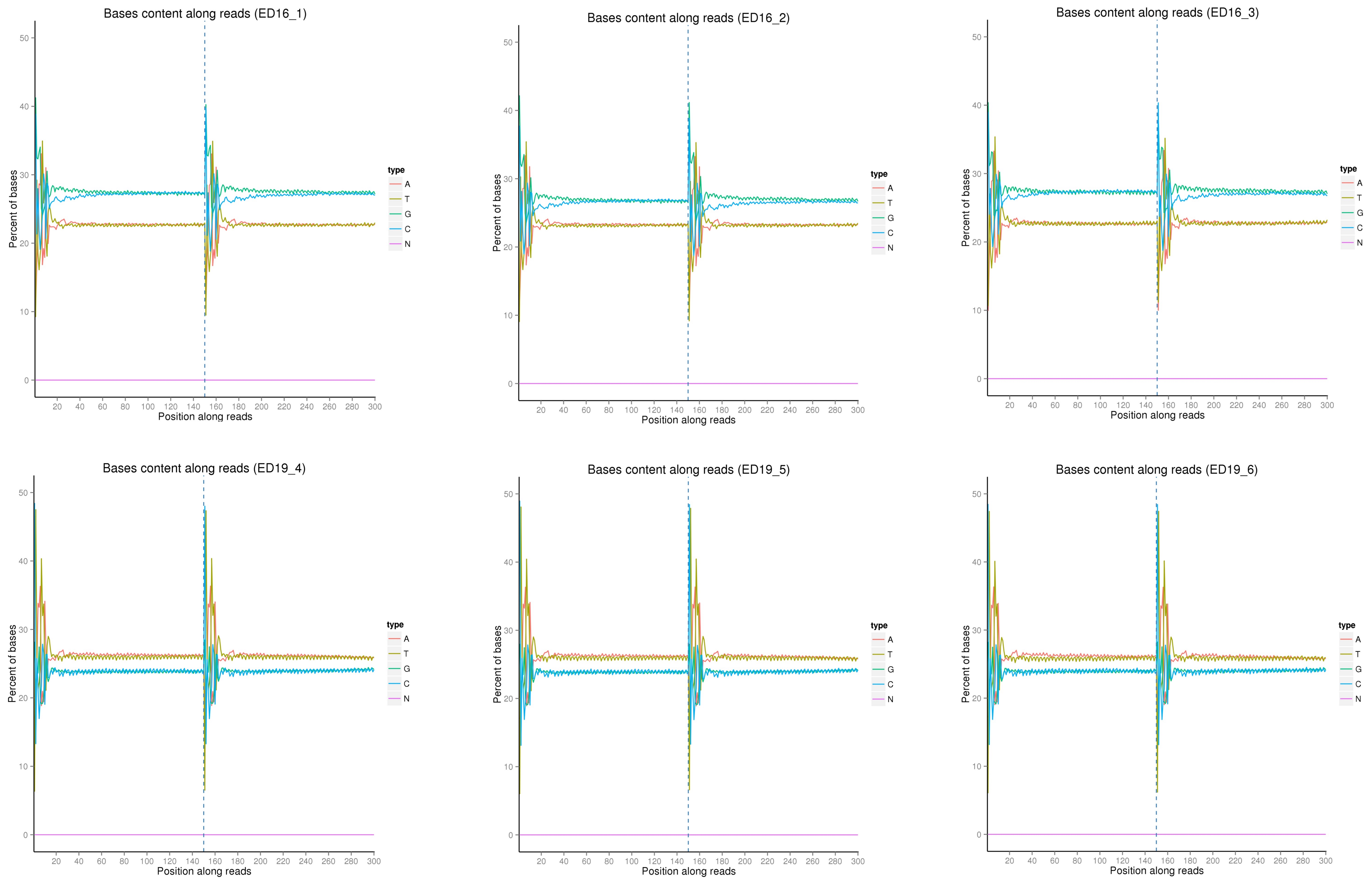


The x-axis shows each base position within a read, and the y-axis shows the percentage of each base, with each base represented by a different color.

**S-Figure 3: Raw Reads Components**


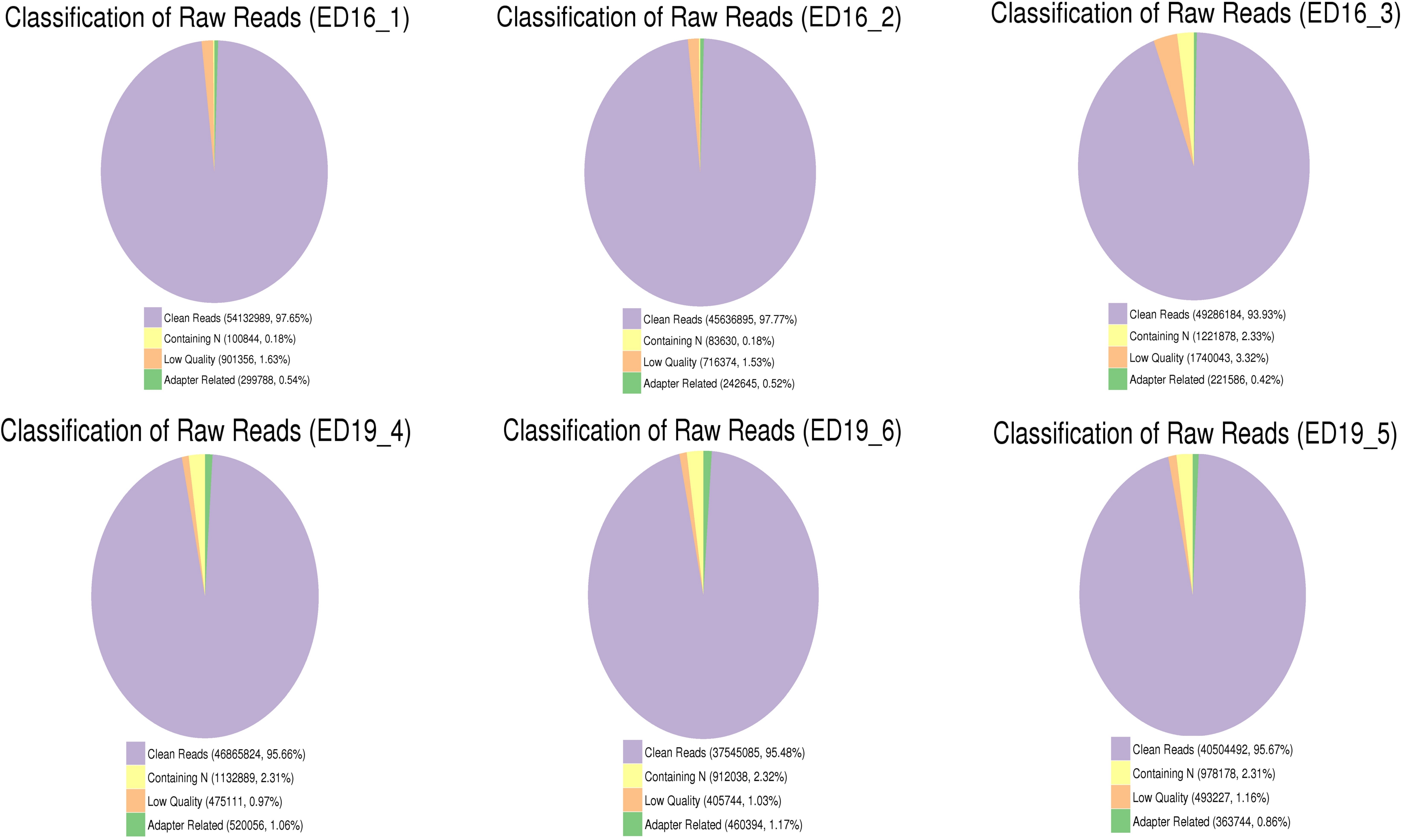


Results are shown as percentage of total raw reads.

(1) Adapter related: reads that had adapter contamination.

(2) Containing N: reads in which uncertain nucleotides constituted more than 10 percent of the read.

(3) Low quality: reads in which low quality nucleotides constituted more than 50 percent of the read.

(4) Clean reads: reads that passed quality control.

**S-Figure 4: Reads Mapped to Reference Genome**


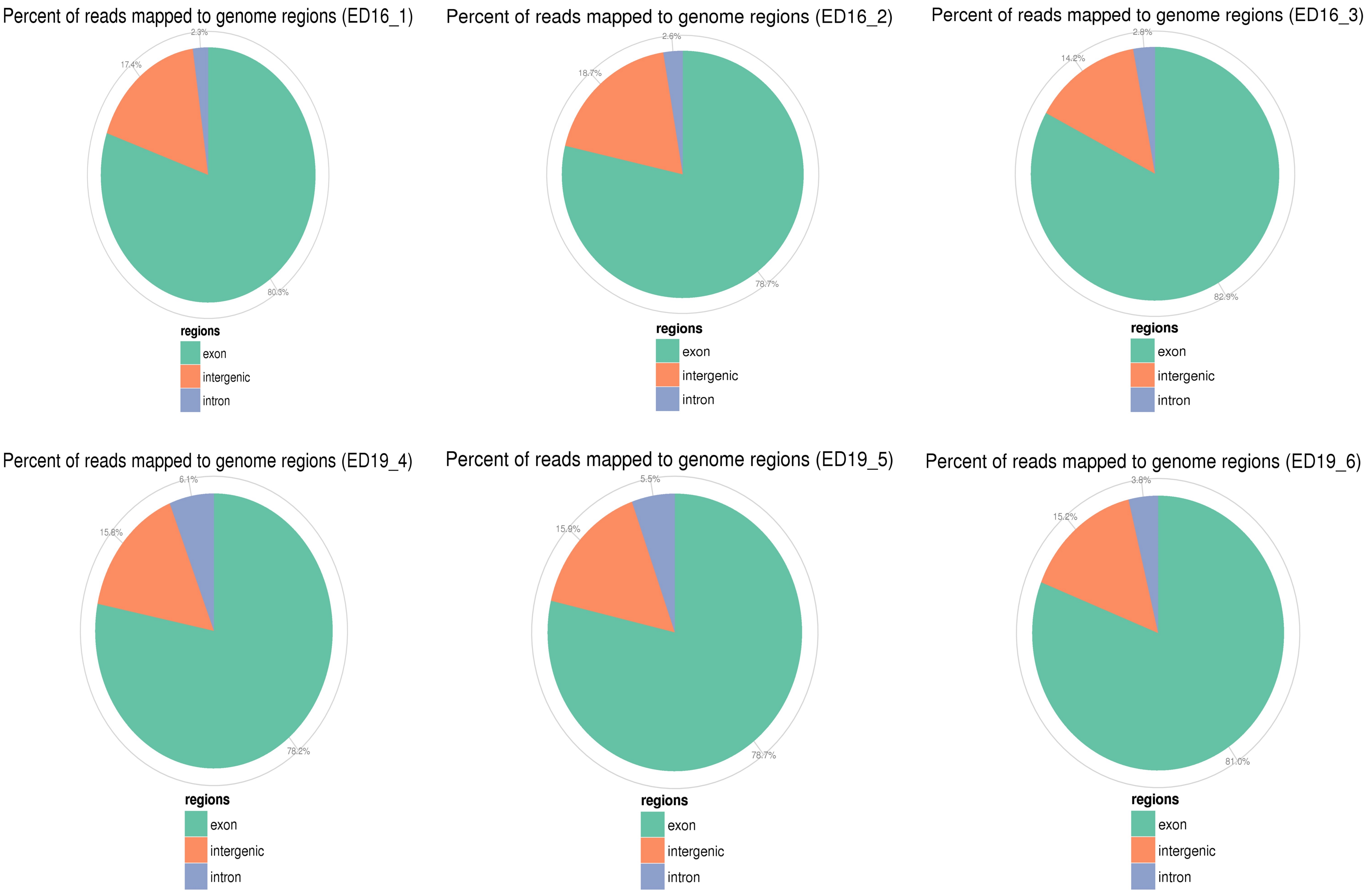


Classification of Reads for each sample are shown here according to Mapped Region. Green color portion represents exon, purple color represents intron and orange color represents intergenic (because of weak annotation of the reference genome).

**S-Figure 5: Distribution of Mapped Reads in Chromosomes**


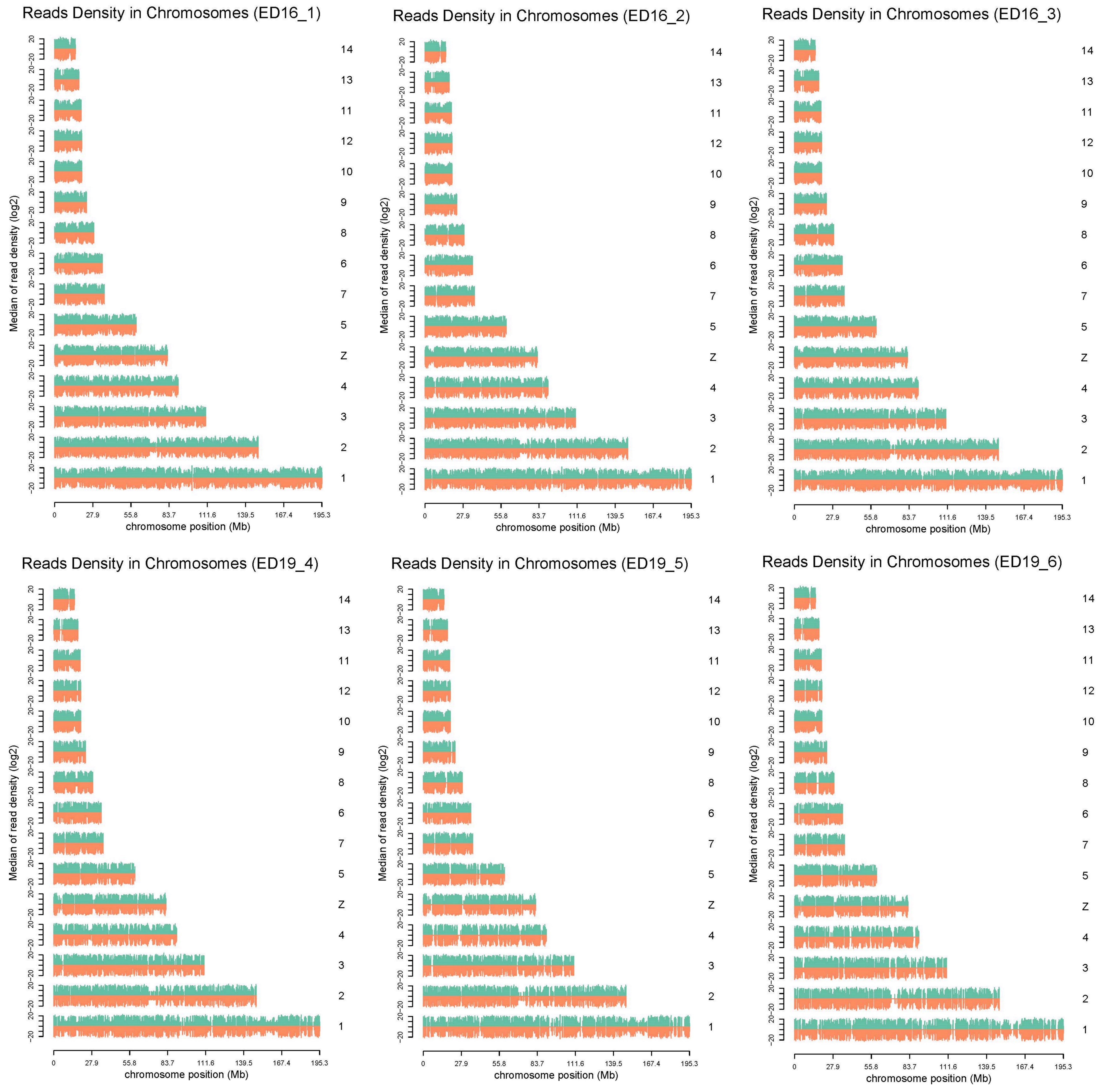


The X-axis shows the length of the chromosomes (in Mb), and the Y-axis indicates the log2 of the median of read density. Green and red indicates the positive and negative strands respectively.

**S-Figure 6: Classification of AS events by rMATS**

**
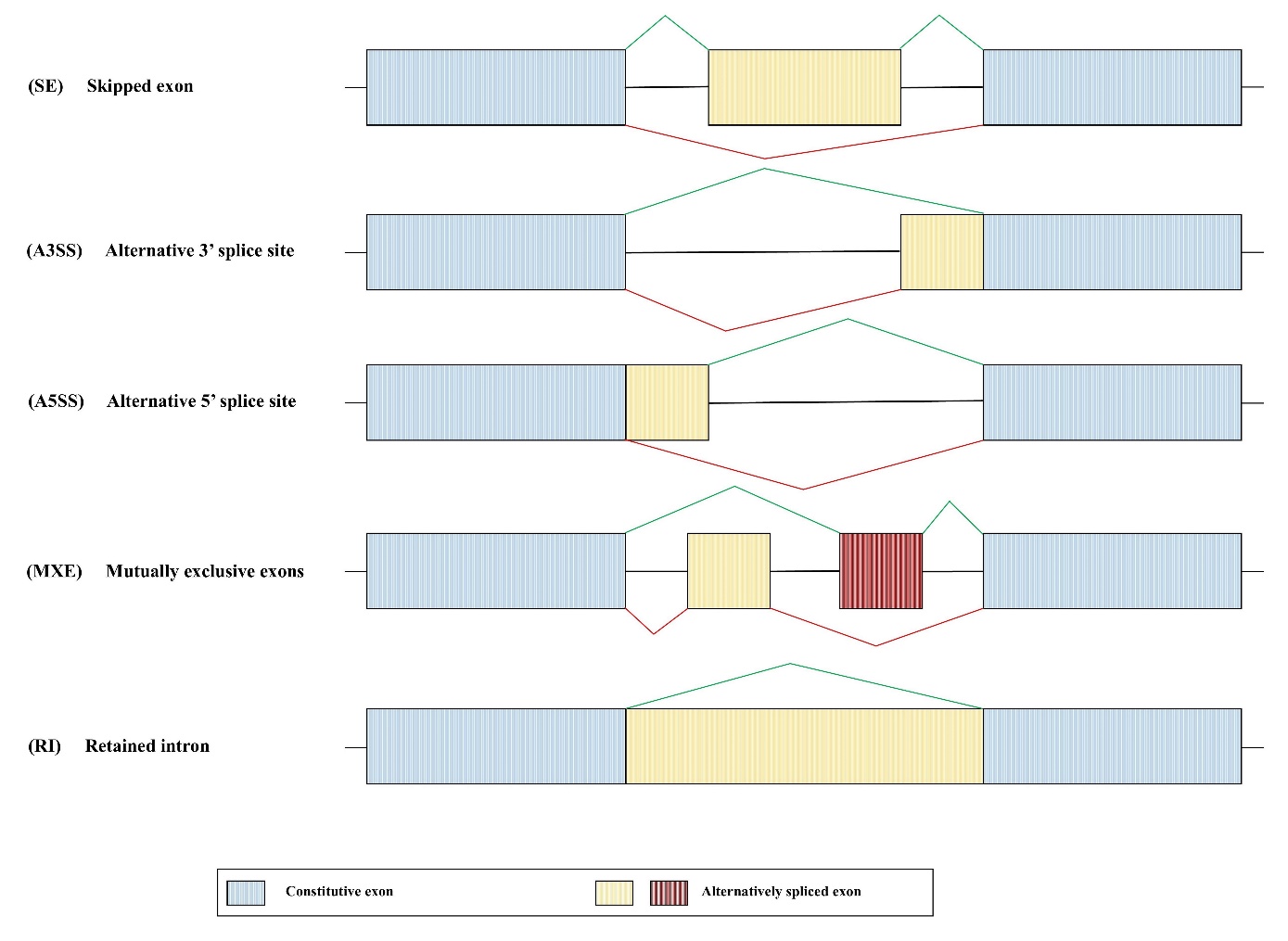
**

The classification of five Alternative splicing (AS) types by rMATS can be defined as: (1) SE: Skipped exon (2) A3SS: Alternative 3’ splice site (3) A5SS: Alternative 5’ splice site (4) MXE: Mutually exclusive exons (5) RI: Retained intron

**S-Figure 7: Classification and statistics of AS events**

**
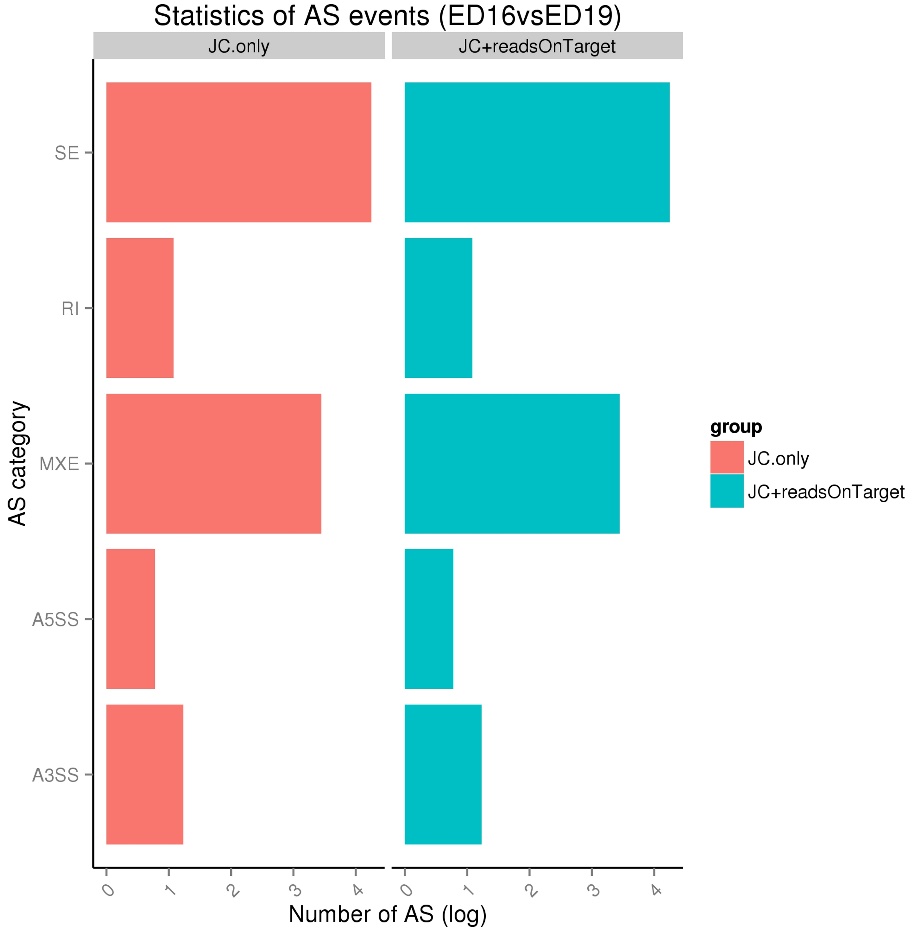
**

The Y-axis illustrates the 5 types of AS events, and the X-axis illustrates the counts for each type of AS events, respectively. JC. only: only the reads span splicing junctions are taken into account; JC+reads On Target: both the reads span splicing junctions and the reads on target are taken into account.

**S-Figure 8: FPKM density distribution for embryonic day 16 and 19 bursal cells from Hy-Line W-36 embryos.**

**
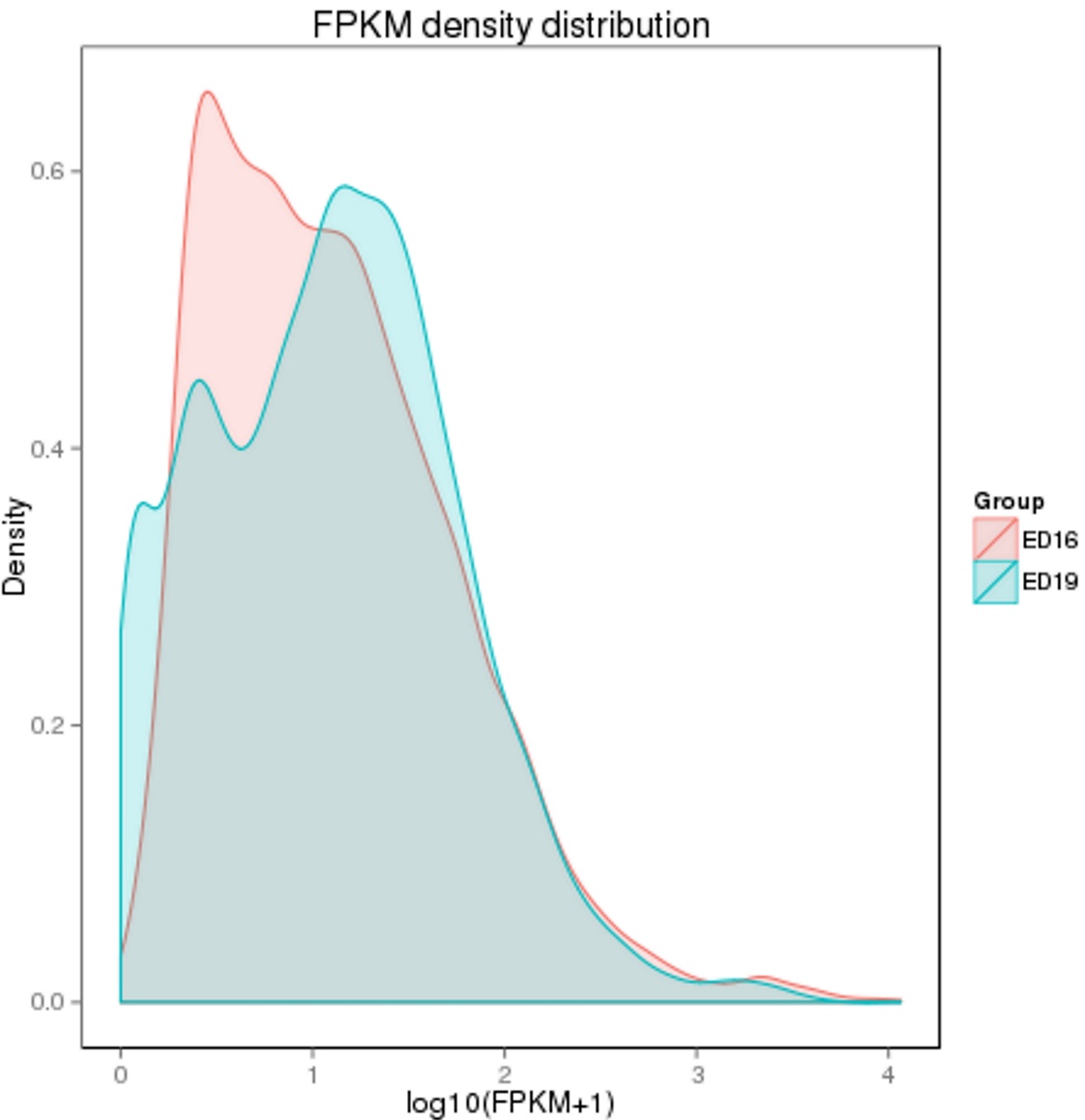
**

FPKM distribution, the x-axis shows the log10(FPKM+1) and the y-axis shows gene density.

**S-Figure 9: FPKM violin distribution for embryonic day 16 and 19 bursal cells from Hy-Line W-36 embryos.**

**
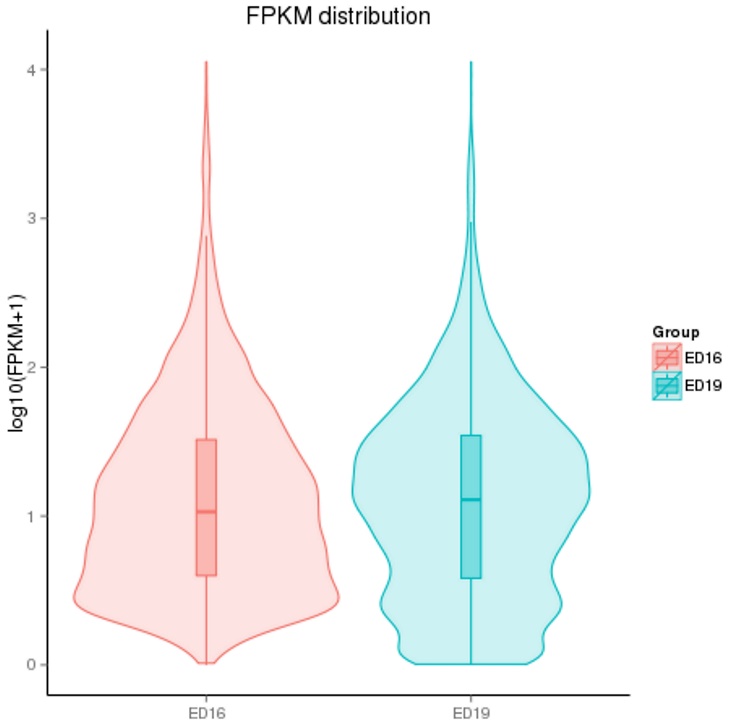
**

FPKM violin Plot, the x-axis shows the group names and the y-axis shows the log10(FPKM+1). Each violin has five statistical magnitudes (max value, upper quartile, median, lower quartile and min value). The violin width shows the gene density.
