# Supplementary figures and images for: Transcriptomic analysis of early B-cell development in the chicken embryo
Source: Poult Sci. 2019 Jun 25;98(11):5342–54. doi: 10.3382/ps/pez354 (PMC6771548; doi:10.3382/ps/pez354)

# Cluster analysis of differentially expressed genes

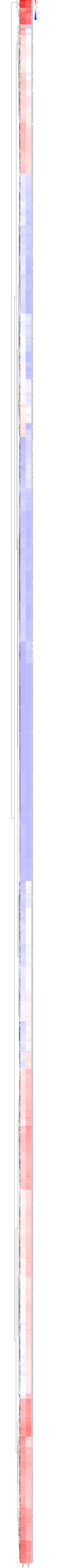

Supplement: pez354_Supplemental_Files [file pez354_supplemental_files.zip › ps-19-08911-S003.pdf]
